# Optical Limiting Performance of Titanium Dioxide-Filled Polyvinylpyrrolidone Composite Nanofibers

**DOI:** 10.1021/acsomega.5c01091

**Published:** 2025-05-28

**Authors:** Yasemin Pepe, Bekir Asilcan Unlu, Serife Akkoyun, Nurcan Asci, Ahmet Karatay, Aytunc Ates, Ayhan Elmali

**Affiliations:** † Department of Engineering Physics, Faculty of Engineering, 37504 Ankara University, Ankara 06100, Türkiye; ‡ Department of Metallurgical and Materials Engineering, Faculty of Engineering and Natural Sciences, 226850 Ankara Yıldırım Beyazıt University, Ankara 06010, Türkiye

## Abstract

In this work, the optical limiting performance of titanium dioxide (TiO_2_) particle-filled polyvinylpyrrolidone (PVP) composite nanofibers produced by electrospinning was investigated. Effectively filled PVP composite nanofibers with TiO_2_ were observed in the backscattering scanning electron microscopy (SEM) images. A reduced band gap energy of the composite nanofibers was determined with an increasing amount of TiO_2_. Increasing localized defect states were obtained with increasing TiO_2_ particles in PVP. Furthermore, open-aperture Z-scan experiments at 532 nm were conducted at different intensities to investigate the composite nanofibers’ nonlinear absorption (NA) characteristics. The NA signal was obtained for all composite nanofibers, and their main NA mechanism was 2PA. The 2PA coefficient and the NA coefficient values of the pure PVP nanofibers increased with the increase in TiO_2_ filler in the PVP nanofibers. This increment indicated the contribution of absorption originating from localized defects inside the NA. Stronger optical limiting performance with a lower onset optical limiting threshold was observed for higher TiO_2_-filled PVP composite nanofibers. TiO_2_ composite nanofibers could be an effective optical limiter in the visible wavelength range due to their strong optical limiting ability, low onset optical limiting threshold, and high optical damage threshold.

## Graphical abstract

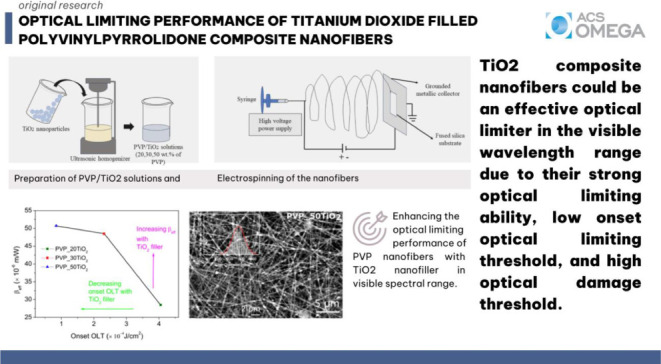

## 1 Introduction

One-dimensional (1D) nanostructured materials like nanotubes, nanowhiskers, nanowires, nanorods, and nanofibers have garnered significant global attention due to their promising technological prospects across various fields such as sensing, photonics, nanoelectronics, and mechanics.^1–3^ Titanium dioxide (TiO_2_) is an n-type semiconductor that has a large energy band gap, high refractive index, outstanding optical transmittance, and chemical stability. The TiO_2_ is a multifunctional substance that is widely acknowledged for its numerous uses, including battery technology, water purification, catalysis, protective coatings, solar energy technology, various sensors (including those for chemical, gas, and biological detection), cosmetics, paint formulations, photovoltaics, CO_2_ reduction, and more.^4–15^ The 1D nanostructured TiO_2_, regarded as one of the most appealing TiO_2_ nanomaterials, has garnered increased attention due to its unique advantages. These include swift electron transport in a singular direction, efficient transfer of charge carriers, and simplicity in constructing nanodevices.^16^ Over recent decades, a range of advanced methodologies, such as electrospinning, solvothermal/hydrothermal processes, electrochemical anodization, templated growth, and chemical vapor deposition, have emerged for the synthesis of one-dimensional (1D) nanostructured TiO_2_ in various forms, including nanotubes, nanobelts, nanorods, nanowires, and nanofibers.^17–19^ Among these methods, when combined with notable attributes like controllable directionality, high porosity, high surface-to-volume ratio, and the capability to integrate multiple components into a single nanofiber, this technique becomes exceptionally potent.^20–22^

Electrospinning is employed to fabricate and assemble nanofiber mats into membranes for various applications, such as biomedicine, protective gear, nanosensors, electronic devices, and clothing with protective functionalities.^23–27^ Nanofibers bear a striking resemblance to many organs and tissues, including skin, collagen, cartilage, and bone, making them suitable for biomedical applications such as tissue engineering, wound dressing, drug delivery systems, and enzyme immobilization.^28^ Nanofibers are also utilized in filtration applications, serving as micro- and nano-filters designed within membranes.^29,30^ While traditional methods for producing antibacterial fabrics have introduced novel applications, the incorporation of nanofibers has provided a feasible enhancement to these methods.^31–33^ Considerable efforts have been directed toward the fabrication of intelligent nanofiber-based sensors, which exhibit both sensing capabilities and the wearable properties typical of conventional textiles.^34,35^

Three distinct crystalline forms of TiO_2_ are known to occur in nature: rutile, anatase, and brookite. The potential uses of anatase and rutile TiO_2_ films in solar cells, self-cleaning coatings, and photocatalysis have been extensively described. The nonlinear optical properties of the rutile and anatase TiO_2_ films have been examined using the third-harmonic generation method.^36^ Large χ^(3)^ values were obtained in nanoporous anatase TiO_2_ films produced by the sol–gel method using a Nd:YAG laser (1064 nm, 42 ps).^37^ Third-order optical nonlinearities of anatase and rutile TiO_2_ thin films were investigated with the closed aperture Z-scan technique using a femtosecond laser system (800 nm, 50 fs), and the findings showed that TiO_2_ films with the anatase phase exhibit larger nonlinear refractive effects in comparison to the rutile phase TiO_2_ films.^38^ The results also revealed that the nonlinear refractive response and nonlinear absorption together contribute to nonlinear optical processes in both phases. Moreover, the physical properties of TiO_2_ are affected not only by the phase but also by the agglomerated microstructure, pores, and particle size. Although there are numerous studies of TiO_2_ nanofibers on photocatalytic activity,^39,40^ biomedical applications,^41^ lithium-ion batteries,^42–44^ supercapacitors,^45,46^ antibacterial activity,^47^ and dye-sensitized solar cells,^48–50^ to our knowledge, there are no studies on the optical limiting characteristics of TiO_2_-filled composite nanofibers produced by electrospinning in the literature. It is believed that nanoparticle-filled composite nanofibers may have characteristics that enable them to be used as efficient optical limiters, given the exceptional qualities of both nanoparticles and nanofibers. In this study, TiO_2_ nanoparticle-filled polyvinylpyrrolidone (PVP) composite nanofibers at various filler concentrations were produced by the electrospinning method. Open-aperture (OA) Z-scan experiments were conducted with varying intensities using a 532 nm wavelength. Their nonlinear absorption and optical limiting characteristics were investigated systematically.

## 2 Materials and Methods

### 2.1 Production of TiO_2_ Composite Nanofibers

TiO_2_ nanoparticles with <25 nm particle size were purchased from Sigma–Aldrich (product number: 637254). Polyvinylpyrrolidone (K85–95) powder was purchased from ACROS Organics. 96% pure ethanol was supplied by Aytaş, Turkey. PVP/ethanol solutions were first prepared by dissolving 7 wt % of PVP powder in ethanol. The solutions were prepared at ambient temperature under constant magnetic stirring. TiO_2_ particles were added to the polymer solutions in various amounts of 20, 30, and 50 wt %. Higher TiO_2_ contents in the PVP solution did not allow for the formation of nanofibers. For better dispersion of the particles, ultrasonic homogenization was applied to the polymer/filler solutions. Then, the solutions were electrospun at a high voltage of 17.5 kV, a tip-to-collector distance of 15 cm, and a flow rate of 1.25 mL/h. Composite nanofibers were collected on a fused silica substrate placed on the metal collector plate for periods of 3, 5, and 10 min. The steps of the process are schematically shown in Figure 1.

**Figure 1:**
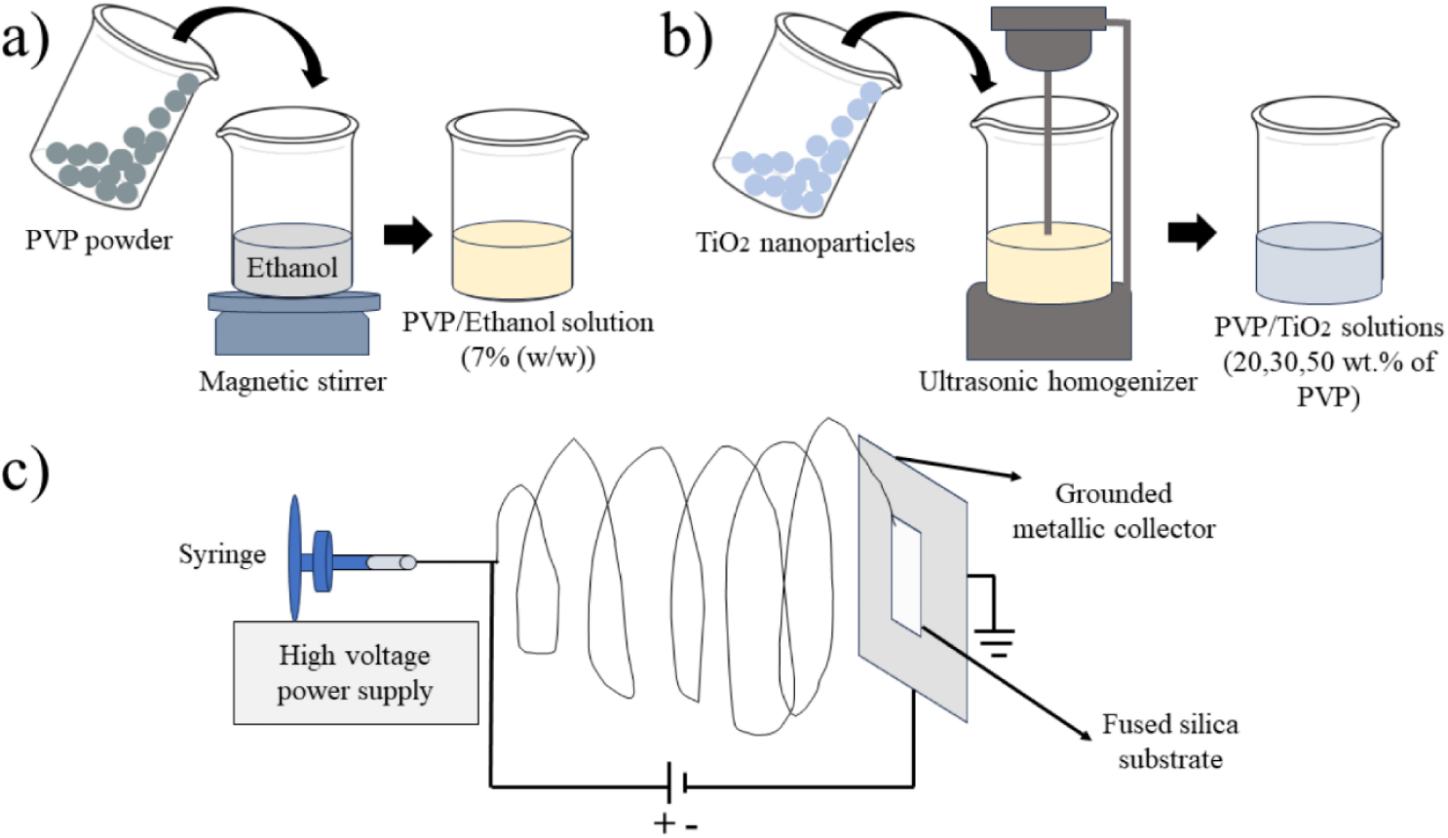
Schematic of the (a) preparation of PVP/ethanol solutions; (b) preparation of PVP/TiO_2_ solutions; and (c) electrospinning of the nanofibers.

### 2.2 Characterization

The morphology of PVP and PVP/TiO_2_ composite nanofibers was examined using a Field-Emission SEM instrument (HITACHI SU5000). Nanofiber diameters were evaluated from SEM micrographs of 100 nanofibers using ImageJ software (NIH-USA). An Oxford X-Max 80 Energy-Dispersive X-ray Spectroscopy (EDS) detector, mounted on the SEM, was used to obtain elemental maps of the samples. All samples were mounted on aluminum (Al) tape and sputter-coated with carbon (C) prior to analysis. Linear optical measurements were conducted by using a UV–vis spectrophotometer (Shimadzu UV-1800). Photoluminescence measurements were carried out with a PerkinElmer LS55 spectrophotometer at an excitation wavelength of 295 nm. The nonlinear optical properties of the composite nanofibers were elucidated through OA Z-scan measurements at 532 nm, employing a Q-switched Nd:YAG laser (Quantel Brilliant) with a pulse duration of 4 ns and a repetition rate of 10 Hz.

## 3 Results and Discussion

### 3.1 Morphological Analysis of TiO_2_ Composite Nanofibers

SEM micrographs of the samples are presented in Figure 2. For better observation, the images are provided in the backscattered electron (BSE) mode, in which the positions of TiO_2_ particles are more discernible. According to the micrographs, PVP and TiO_2_ composite nanofibers have a cylindrical morphology. Additionally, a decrease in average fiber diameters was observed with increasing filler content, as shown in Table 1. PVP nanofibers with 50 wt % TiO_2_ filler exhibit the highest decrease in diameter when compared to PVP nanofibers. A similar trend has been reported in the literature.^51,52^ In the study by Bakhsheshi-Rad et al.,^52^ the use of silk sericin (SS)-filled poly­(vinyl alcohol) (PVA)/chitosan (CS) electrospun nanofibers as wound dressing was investigated. According to the results, the fiber diameters of PVA/CS/SS nanofibers decreased with increasing silk protein concentration. Huang et al. attributed this change in fiber diameter to the increase in the surface charge of the polymer jet owing to the higher electrical conductivity of the filler nanoparticles compared to the polymer solution. As the filler content rises, the polymer jet experiences strong extensional forces, which cause the nanofibers to become thinner. Moreover, as can be seen in closer images shown as insets in Figure 2, TiO_2_ particles are well entrapped inside the nanofibers for each filler content. Additionally, a few TiO_2_ aggregates also appear in different regions of the PVP_50TiO_2_ sample. EDS maps provided in Figure 3 further support these findings. In addition to the presence of carbon (C), oxygen (O), and nitrogen (N), which are the main constituent elements of the PVP polymer, the presence of titanium (Ti), especially located on the nanoparticles entrapped inside the nanofibers, has been established. The presence of aluminum (Al) is ascribed to the tape.

**Figure 2:**
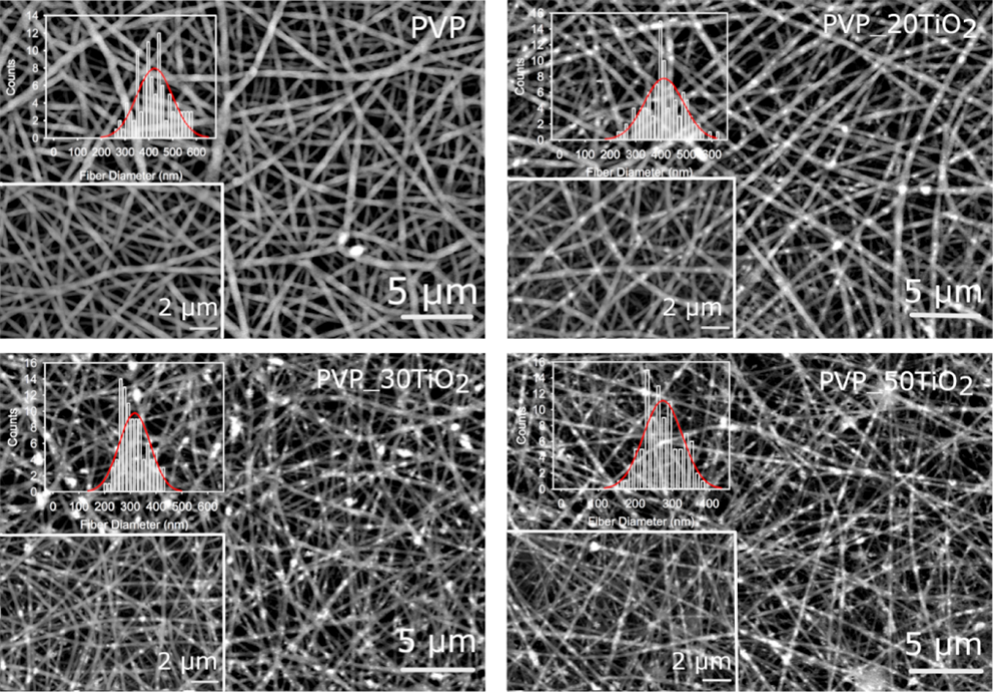
SEM and BSE micrographs of the composite nanofibers.

**Table 1:** Fiber Diameters of PVP and PVP/TiO_2_ Nanofibers

Nanofibers	Average fiber diameters (nm)
PVP	424 ± 75.0
PVP_20TiO_2_	411 ± 77.0
PVP_30TiO_2_	317 ± 60.6
PVP_50TiO_2_	275 ± 52.6

**Figure 3:**
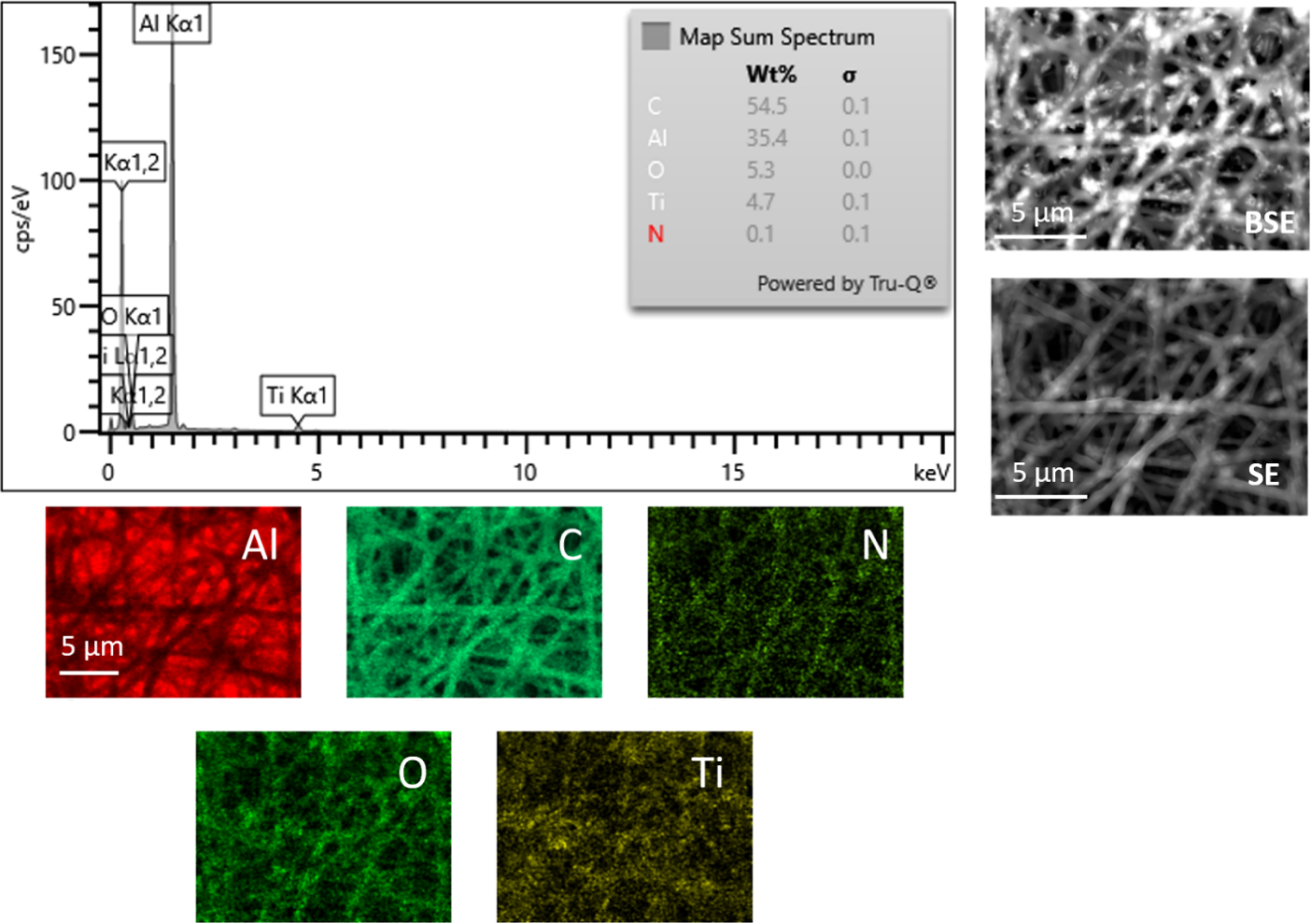
EDS spectrum and elemental maps of PVP/TiO_2_ nanofibers. BSE and SE images of the analysis area are also provided.

### 3.2 Linear Optical Analysis of TiO_2_ Composite Nanofibers

Linear absorption spectra of pure PVP and TiO_2_ composite nanofibers are presented in Figure 4. It is clearly seen in this figure that the TiO_2_ filler led to enhanced linear absorption, and the linear absorption increased with an increase in filler concentration. The strong absorption at 295 nm was observed for pure PVP nanofibers, and it slightly shifted to 300 nm and expanded to the near-infrared region with an increase in the TiO_2_ filler concentration. To examine the effective electron transfer mechanisms in the studied nanofibers, the equation provided in ref.^53^ was utilized, and their Tauc plots are given in Figure 4 b. The band gap energy was found to be 4.06 eV for pure PVP nanofibers, and it decreased to 3.98 eV with TiO_2_ filler. Diminishing the particle size induces quantum confinement for both electrons and holes in all three dimensions, consequently increasing the effective band gap of the material. Conversely, particle agglomeration leads to a decreased band gap energy. Hence, the reduced band gap energy observed in the composite nanofibers can be attributed to the agglomerated TiO_2_ nanoparticles within the PVP nanofibers.

**Figure 4:**
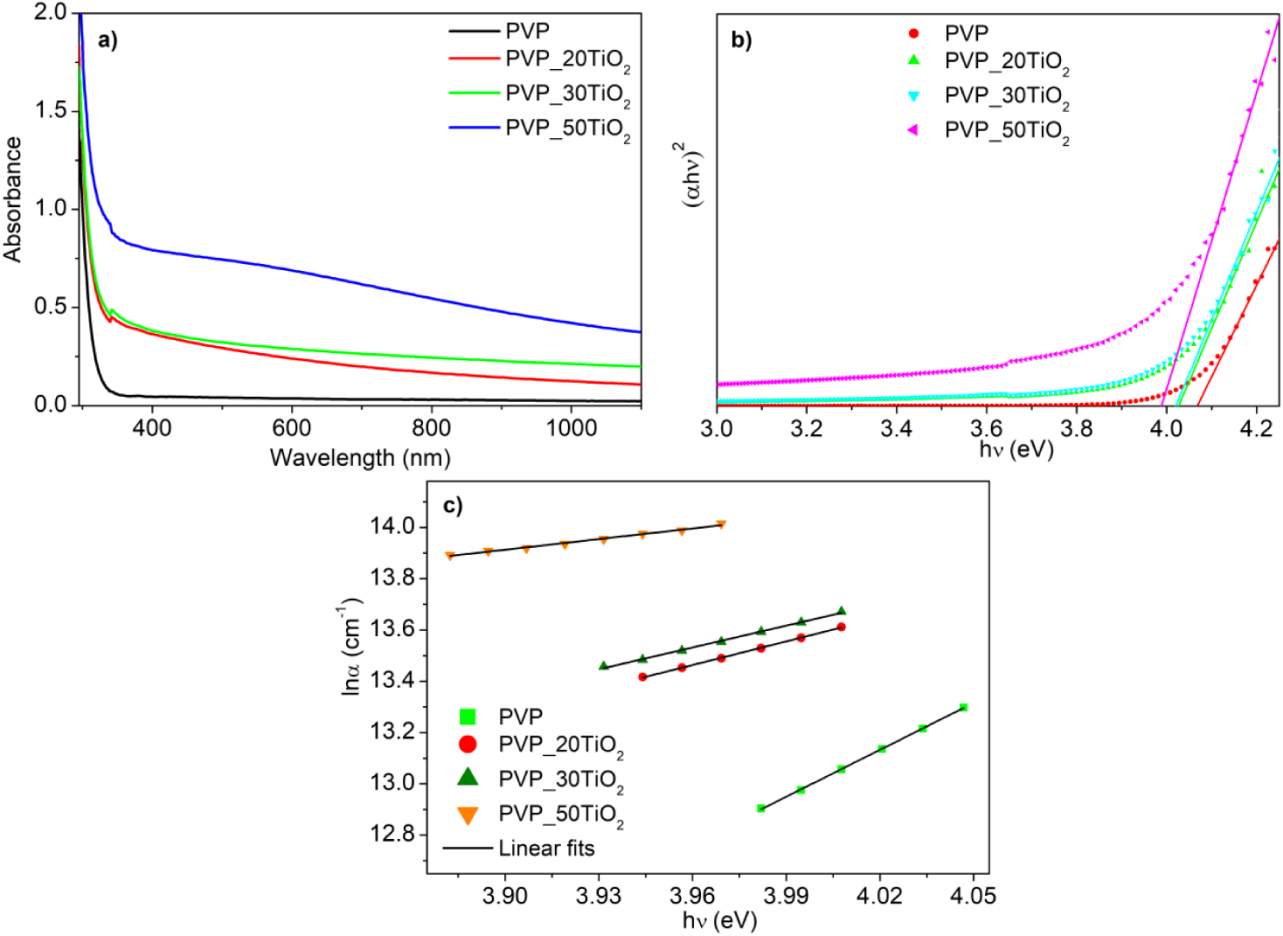
Absorbance spectra, (b) Tauc plots graphs ((*αhν*)^2^ versus *hν*), and (c) ln*α* versus *hν* plot of pure PVP nanofibers and TiO_2_ composite nanofibers.

The characterization of band gap energy, alongside the density and distribution of defect states within the band gap, plays a vital role in elucidating nonlinear optical properties. The material’s Urbach energy, which provides information about defect states within the band gap, is derived from the width of the tail edge of the absorption band, also known as the Urbach tail. The Urbach energy of the samples was determined using the following expression:^54^

1 α=α0exp(hvEU)

where *α* is the absorption coefficient, *α*_0_ is a constant, and *E*_*U*_ is the Urbach energy. The *E*_*U*_ values of the samples were found from the inverse slope of the ln*α* versus *hν* plots presented in Figure 4 c.

The corresponding *E*_*U*_ values of pure PVP nanofibers, PVP_20TiO_2_, PVP_30TiO_2_, and PVP_50TiO_2_ composite nanofibers were found to be 0.16, 0.32, 0.36, and 0.72 eV, respectively. The defect levels corresponding to these energies are localized just below the conduction band because the Urbach energies are obtained from the absorption band edge thickness. Moreover, this result revealed the increased localized defect states with an increase in TiO_2_ filler concentration in PVP nanofibers. According to this result, the PVP_50TiO_2_ composite nanofibers had more defect states compared with other composite nanofibers. As the presence of defects significantly influences electron transfer mechanisms, which are related to nonlinear absorption, it is important to ascertain the spatial arrangement of defect levels across the energy band gap. For this purpose, the photoluminescence spectra acquired with a 295 nm excitation wavelength for both pure PVP and composite nanofibers were examined and are depicted in Figure 5. As shown in the figure, all the studied nanofibers demonstrate broad emission between 340 and 650 nm. This broad emission indicated that localized defect states are widely distributed across the band gap. The emission peaks localized at about 384, 422, 486, and 531 nm were observed. The prominent blue emission at 384 nm can be attributed to electron–hole recombination, while the remaining emission peaks can be attributed to surface trap-induced emission caused by the presence of PVP.^55^ The intensity of these peaks increased with an increase in the TiO_2_ filler concentration within PVP nanofibers.

**Figure 5:**
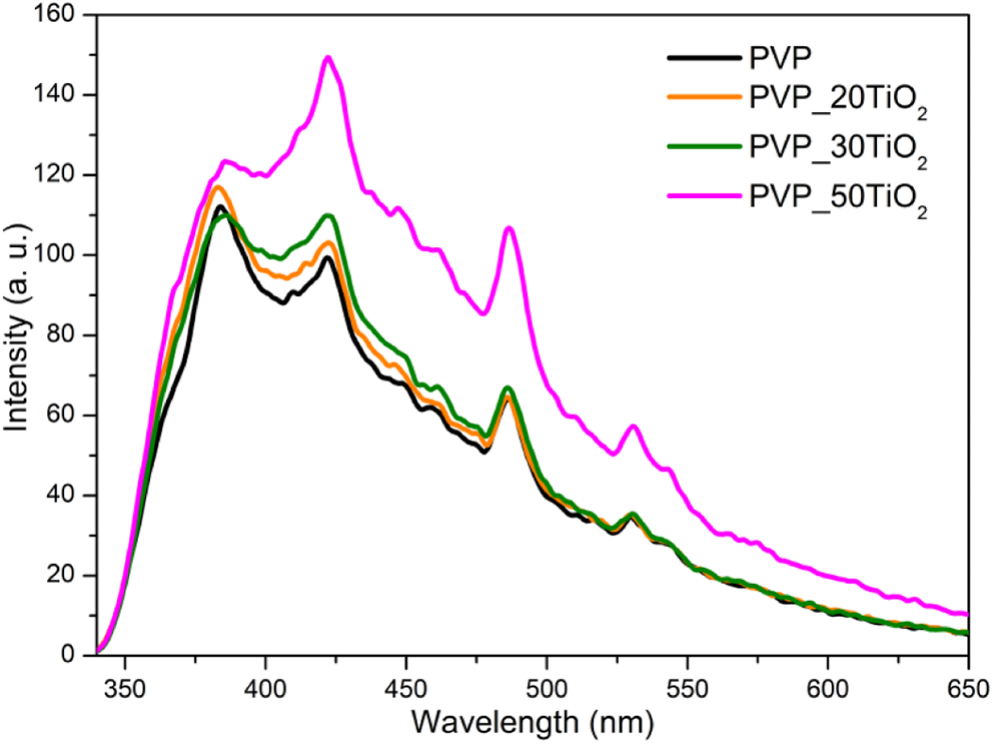
Photoluminescence spectra of pure PVP and TiO_2_ composite nanofibers.

### 3.3 **Nonlinear Absorption and Optical Limiting Analysis of TiO**_**2**_ **Composite Nanofibers**

The OA Z-scan measurements were performed at 532 nm under various input intensities to reveal the nonlinear absorption (NA) characteristics of the composite nanofibers. Figure 6 shows the OA Z-scan curves of the studied nanofibers. All of the nanofibers exhibit NA features, and the pure PVP nanofibers’ NA behavior became weaker with increasing input intensities, as seen in Figure 6 a. Considering Figure 5, it displays an emission band at approximately one photon energy (∼2.32 eV). The weakening of the NA with increasing intensity can be attributed to the filling of these localized defect states by one-photon absorption (1PA). On the other hand, the composite nanofibers’ NA behavior became stronger with an increase in the input intensity, as observed in Figure 6b,c,d. To determine the two-photon absorption (2PA) coefficient of the studied nanofibers, the OA Z-scan curves were fitted using the 2PA procedure introduced by Sheik-Bahae et al.^56^

2 T(z)=∑m=0∞q0(m+1)3/2(1+z2z02)m

**Figure 6:**
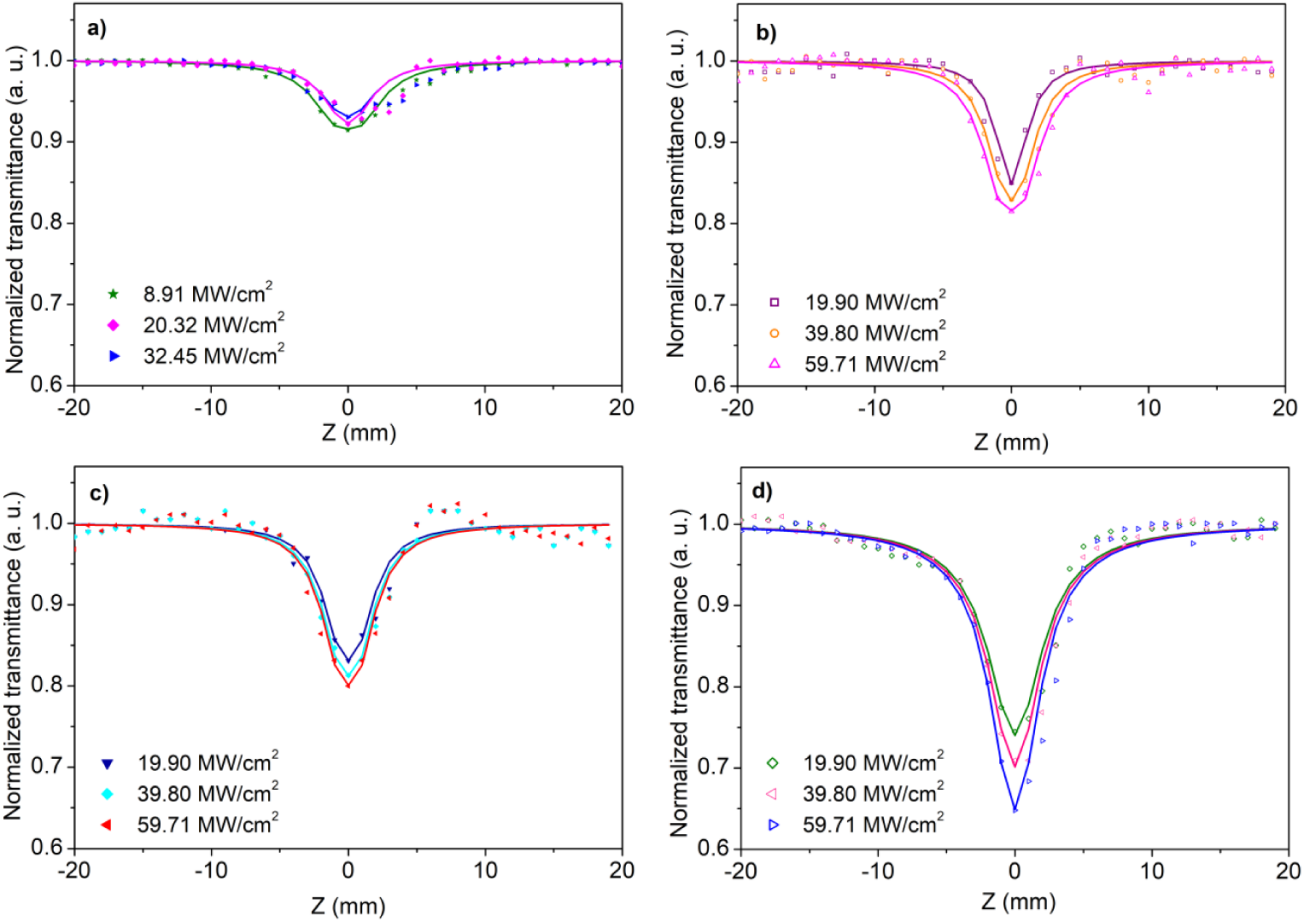
Open aperture Z-scan curves of (a) pure PVP nanofibers, (b) PVP_30TiO_2_, (c) PVP_20TiO_2_, and (d) PVP_50TiO_2_ composite nanofibers under various input intensities.

where *m* is an integer, *q*_0_(*z*) is *βI*_0_ *L*_eff_, *β* is the 2PA coefficient, and the effective thickness is $L_{\mathrm{eff}}=\frac{1-e^{-\alpha_{0}L}}{\alpha_{0}}$ with equal to the sample thickness *L*, and *α*_0_ is the linear absorption coefficient. The values of *β* are listed in Table 2. As shown in the table, the *β* value of the pure PVP nanofibers decreased from 4.20 × 10^–7^ m/W to 2.40 × 10^–7^ m/W with an increase in the input intensity. TiO_2_ composite nanofibers exhibited higher *β* values compared to pure PVP nanofibers, with the highest *β* value obtained for PVP_50TiO_2_ compared to other composite nanofibers. This result provides evidence that the contribution of TPA to NA increases with increasing input density. As reported in the previous section, the TiO_2_ composite nanofibers had a more defective structure compared to the pure PVP nanofibers. It has been extensively reported that increased localized defect states lead to enhanced NA behavior in various materials.^57–61^ To investigate the influence of localized defect states on NA behavior, a theoretical model was used to obtain the nonlinear absorption coefficient (*β*_eff_) and saturable intensity threshold (*I*_SAT_) of the studied samples. In this model, the 1PA, 2PA, and free carrier absorption (FCA) contributions to NA were considered. The details of the fitting can be found elsewhere.^62^

3 dIdz=αI1+IISAT−βI21+I2ISAT2−σ0ΔN(I)I1+I2ISAT2

**Table 2:** Nonlinear Absorption Coefficients (*β*_eff_), 2PA Coefficients (*β*), Saturation Intensity Threshold (*I*_SAT_), and Onset Optical Limiting Threshold (OLT) Values of Pure PVP and TiO_2_ Composite Nanofibers

Samples	PVP			PVP_20TiO_2_			PVP_30TiO_2_			PVP_50TiO_2_		
*I*_0_ (MW/cm^2^)	8.52	27.86	49.76	19.90	39.80	59.71	19.90	39.80	59.71	19.90	39.80	59.71
*β* (×10^–7^ m/W)	4.20	3.52	2.40	6.05	7.49	15.5	6.43	8.29	18.6	13.89	15.90	19.13
*β*_eff_ (×10^–6^ m/W)	10.4	4.85	0.62	14.1	17.2	28.5	18.2	20.6	48.5	19.6	25.7	50.7
*I*_SAT_ (×10^13^ W/m^2^)	0.78	0.45	0.13	1.82	9.16	21.5	1.94	37.20	40.60	16.6	40.02	74.00
Onset OLT (×10^–4^ J/cm^2^)						4.05			2.31			0.84

Δ*N*(*I*) is the generated photocarrier density, and *β*_eff_ is the effective NA coefficient given as

4 $$\Delta N=\frac{\alpha I}{\hbar \omega }\tau_{0}$$

5 $$\beta_{\mathrm{eff}}=\beta +\left(\sigma_{0}\alpha \tau_{0}/\hbar \omega \right)$$

where *α* is the linear absorption coefficient, ℏ*ω* is the photon energy, *β* is the 2PA coefficient, *τ*_0_ is the pulse duration, and *σ*_0_ is the FCA cross section. The following expression can be obtained by substituting eqs 4and[Disp-formula eq5] in eq 3. The beam waist at the focus and the Rayleigh length of the composite nanofibers are 24 μm and 0.34 cm, respectively.

6 dIdz′=−αI1+IISAT−βeffI21+I2ISAT2

The obtained *β*_eff_ and *I*_SAT_ values of the studied samples are presented in Table 2. The *β*_eff_ value of the pure PVP nanofibers was found to be 10.4 × 10^–6^ m/W and decreased to 0.62 × 10^–6^ m/W with increasing input intensity. This decrease may be attributed to the reduced ground-state absorption with increasing input intensity. On the other hand, the *β*_eff_ values of the composite nanofibers were higher than those of the pure PVP nanofibers, and they increased with the rise in input intensity. The *β*_eff_ value of the pure PVP nanofibers increased from 0.62 × 10^–6^ m/W to 28.5, 48.5, and 50.7 × 10^–6^ m/W with increasing TiO_2_ filler concentration in the PVP nanofibers. Among the composite nanofibers, the PVP_50TiO_2_ composite nanofibers had stronger NA behavior. Considering the results reported in the section below section, the PVP_50TiO_2_ composite nanofibers had a more defective structure. This finding indicates the influence of localized defect states on NA behavior. Increased localized defect states with TiO_2_ filler concentration in PVP nanofibers led to additional absorption mechanisms, which resulted in enhanced NA behavior. Similarly, the *I*_SAT_ values of the composite nanofibers were higher than those of the pure PVP nanofibers, and they increased with the rise in input intensity. This result further supports the enhanced NA behavior. Compared to *β* and *β*_eff_ values, the *β*_eff_ values of the composite nanofibers were almost 23 times higher than their β values. This result suggests that the localized defect states created by the TiO_2_ filler in PVP contributed significantly to NA behavior. The primary NA mechanism of TiO_2_ composite nanofibers was sequential 2PA. The NA coefficients from some reported studies are tabulated in Table 3 for comparison with the present results. The NA coefficients of the present composite nanofibers are higher than those listed in Table 3.

**Table 3:** NA Coefficients of Several Nonlinear Optical Materials

Samples	β (m/W)	ref.
TiO_2_ colloids (7 ns, 532 nm)	77.8 × 10^–11^	^63^
ZnO–TiO_2_ nanocomposite (7 ns, 532 nm)	18 × 10^–10^	^63^
TiO_2_ nanoparticles (150 fs, 680 nm)	5.1 × 10^–10^	^64^
C–N–S-doped TiO_2_ nanoparticles (9 ns, 532 nm)	1.81 ± 0.18 × 10^–10^	^65^
PVA/(*x*)TiO_2_ (15 – *x*) CuO nanocomposites (cw, 532 nm)	8.17 × 10^–6^	^66^
GO-TiO_2_ composites (6 ns, 532 nm)	127 × 10^–10^	^67^

In recent years, lasers have garnered extensive applications across diverse sectors, including the processing industry, scientific research, and medical and defense domains, owing to their attributes such as high power, coherence, and brightness. The next emerging problem concerns the protection of sensitive components such as eyes, sensors, photodetectors, and photoelectric devices. For this, there is an urgent need for optical limiters that block high-energy beams to prevent damage caused by sudden laser exposure. Nonlinearly absorbing materials are typically the best candidates for self-adaptive optical limiters because they permit low-energy light to flow through while blocking the transmission of high-energy light. To obtain the onset optical limiting threshold (OLT) of the composite nanofibers, the fluence values in the samples were calculated, and the corresponding optical limiting curves are depicted in Figure 7. The fluence level at which the normalized transmittance initiates a decrease relative to the fluence was designated as the onset of the OLT values. A low OLT and high damage threshold are required for efficient optical limiters. The onset OLT values were obtained as 4.05 × 10^–4^, 2.31 × 10^–4^, and 0.84 × 10^–4^ for PVP_20TiO_2_, PVP_30TiO_2_, and PVP_50TiO_2_ composite nanofibers, respectively. The PVP_50TiO_2_ composite nanofibers showed stronger optical limiting due to their stronger NA behavior compared with other composite nanofibers. The laser damage threshold values of the TiO_2_ composite nanofibers were obtained between 0.25 and 0.28 J/cm^2^, which are larger than that of CsPbBr_3_ (∼32 mJ/cm^2^).^68^ Seetharaman et al. investigated the optical limiting behavior of the C–N–S-doped TiO_2_ nanoparticles and reported a value of 1.92 J/cm^2^.^65^ Mariserla et al. reported the OLT of the GO-TiO_2_ composites as 0.55 J/cm^2^.^67^ These reported values are much higher than those of TiO_2_ composite nanofibers. The strong optical limiting behavior with a low onset OLT value and high laser damage threshold has made TiO_2_ composite nanofibers a promising candidate as an efficient optical limiter in the visible wavelength region.

**Figure 7:**
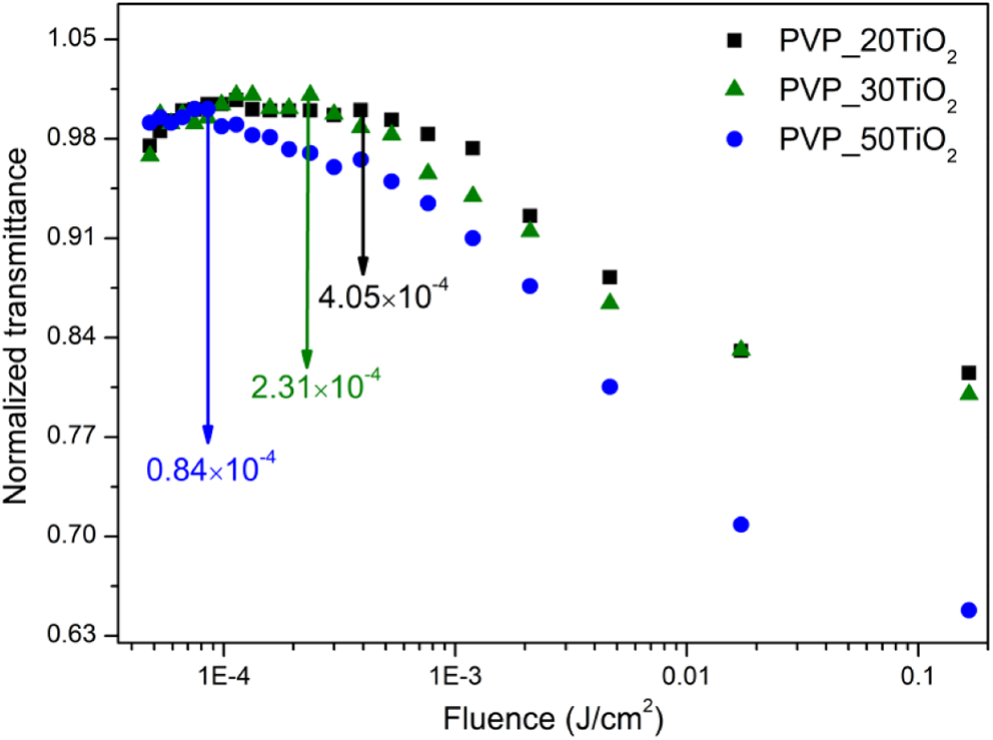
Optical limiting curves of the TiO_2_ composite nanofibers.

## 4 Conclusion

In this work, the optical limiting capabilities of TiO_2_ nanoparticle-filled PVP composite nanofibers were investigated. The SEM images of the composite nanofibers revealed that TiO_2_ nanoparticles effectively filled the PVP nanofibers. It was observed that the band gap energy of the pure PVP nanofibers reduced from 4.06 to 3.98 eV with an increasing amount of TiO_2_ in the PVP nanofibers. The Urbach energy of the pure PVP nanofibers increased from 0.16 to 0.72 eV with increasing filler concentration in the PVP nanofibers. To examine the NA features of the composite nanofibers, the OA Z-scan experiments were performed at a 532 nm wavelength with varying input intensities. All of the TiO_2_ composite nanofibers exhibited NA behaviors, and the main NA mechanism was 2PA. Their 2PA coefficients and NA coefficients were determined from the fitting of Z-scan data. The 2PA coefficient and the NA coefficient values of the pure PVP nanofibers increased from 2.40 × 10^–7^ m/W to 19.13 × 10^–7^ m/W, and 0.62 × 10^–6^ m/W to 50.7 × 10^–6^ m/W with an increase in the TiO_2_ filler amount in the PVP nanofibers, respectively. This increment indicated the contribution of the absorption originating from localized defects inside NA. The lowest onset of the OLT value was obtained as 0.84 × 10^–4^ J/cm^2^ for PVP_50TiO_2_ among other composite nanofibers. The strong optical limiting performance with a low onset of the OLT and a high optical damage threshold highlights the potential of the TiO_2_ composite nanofibers as a capable optical limiter in the visible spectrum.
